# Supplementary material for: Fine mapping epitope on Glycoprotein-Gn from Severe Fever with Thrombocytopenia Syndrome Virus
Source: PLoS One. 2021 Mar 2;16(3):e0248005. doi: 10.1371/journal.pone.0248005 (PMC7924767; doi:10.1371/journal.pone.0248005)
Supplement: S1 Table — (DOC) [file pone.0248005.s001.doc]

**S1 Table.** 16mer peptides amino acid sequence and its location on SFTSV-Gn.

| Peptide items | Position in Gn | Amino acids | Peptide items | Position in Gn | Amino acids |
| --- | --- | --- | --- | --- | --- |
| P1 | **Gn189-204** | TFLELKSFSQSEFPDI | P9 | **Gn253-268** | KDFVCYKEGTGPCSES |
| P2 | **Gn197-212** | SQSEFPDICKIDGIVF | P10 | **Gn261-276** | GTGPCSESEEKTCKTS |
| P3 | **Gn205-220** | CKIDGIVFNQCEGESL | P11 | **Gn269-284** | EEKTCKTSGSCRGDMQ |
| P4 | **Gn213-228** | NQCEGESLPQPFDVAW | P12 | **Gn277-292** | GSCRGDMQFCKVAGCE |
| P5 | **Gn221-236** | PQPFDVAWMDVGHSHK | P13 | **Gn285-300** | FCKVAGCEHGEEASEA |
| P6 | **Gn229-244** | MDVGHSHKIIMREHKT | P14 | **Gn293-308** | HGEEASEAKCRCSLVH |
| P7 | **Gn237-252** | IIMREHKTKWVQESSS | P15 | **Gn301-316** | KCRCSLVHKPGEVVVS |
| P8 | **Gn245-260** | KWVQESSSKDFVCYKE | P16 | **Gn309-324** | KPGEVVVSYGGMRVRP |
