# Supplementary material for: Fine mapping epitope on Glycoprotein-Gn from Severe Fever with Thrombocytopenia Syndrome Virus
Source: PLoS One. 2021 Mar 2;16(3):e0248005. doi: 10.1371/journal.pone.0248005 (PMC7924767; doi:10.1371/journal.pone.0248005)
Supplement: S2 Table — (DOC) [file pone.0248005.s002.doc]

**S2 Table.** 8/10mer peptides amino acid sequence and its location on SFTSV-Gn.

| positive 16mer peptides | P1 | P6 | P9 | P12 | P13 | P16 | P9 |
| --- | --- | --- | --- | --- | --- | --- | --- |
|  | **Gn189-204** | **Gn229-244** | **Gn253-268** | **Gn277-292** | **Gn285-300** | **Gn309-324** | **Gn253-268** |
| Corresponding 8/10mer peptides | FLELKSFS | DVGHSHKI | DFVCYKEG | SCRGDMQF | CKVAGCEH | PGEVVVSY | KDFVCYKEGT |
|  | LELKSFSQ | VGHSHKII | FVCYKEGT | CRGDMQFC | KVAGCEHG | GEVVVSYG | DFVCYKEGTG |
|  | ELKSFSQS | GHSHKIIM | VCYKEGTG | RGDMQFCK | VAGCEHGE | EVVVSYGG | FVCYKEGTGP |
|  | LKSFSQSE | HSHKIIMR | CYKEGTGP | GDMQFCKV | AGCEHGEE | VVVSYGGM | VCYKEGTGPC |
|  | KSFSQSEF | SHKIIMRE | YKEGTGPC | DMQFCKVA | GCEHGEEA | VVSYGGMR | CYKEGTGPCS |
|  | SFSQSEFP | HKIIMREH | KEGTGPCS | MQFCKVAG | CEHGEEAS | VSYGGMRV | YKEGTGPCSE |
|  | FSQSEFPD | KIIMREHK | EGTGPCSE | QFCKVAGC | EHGEEASE | SYGGMRVR |  |
