# Supplementary figures and images for: Fine mapping epitope on Glycoprotein-Gn from Severe Fever with Thrombocytopenia Syndrome Virus
Source: PLoS One. 2021 Mar 2;16(3):e0248005. doi: 10.1371/journal.pone.0248005 (PMC7924767; doi:10.1371/journal.pone.0248005)

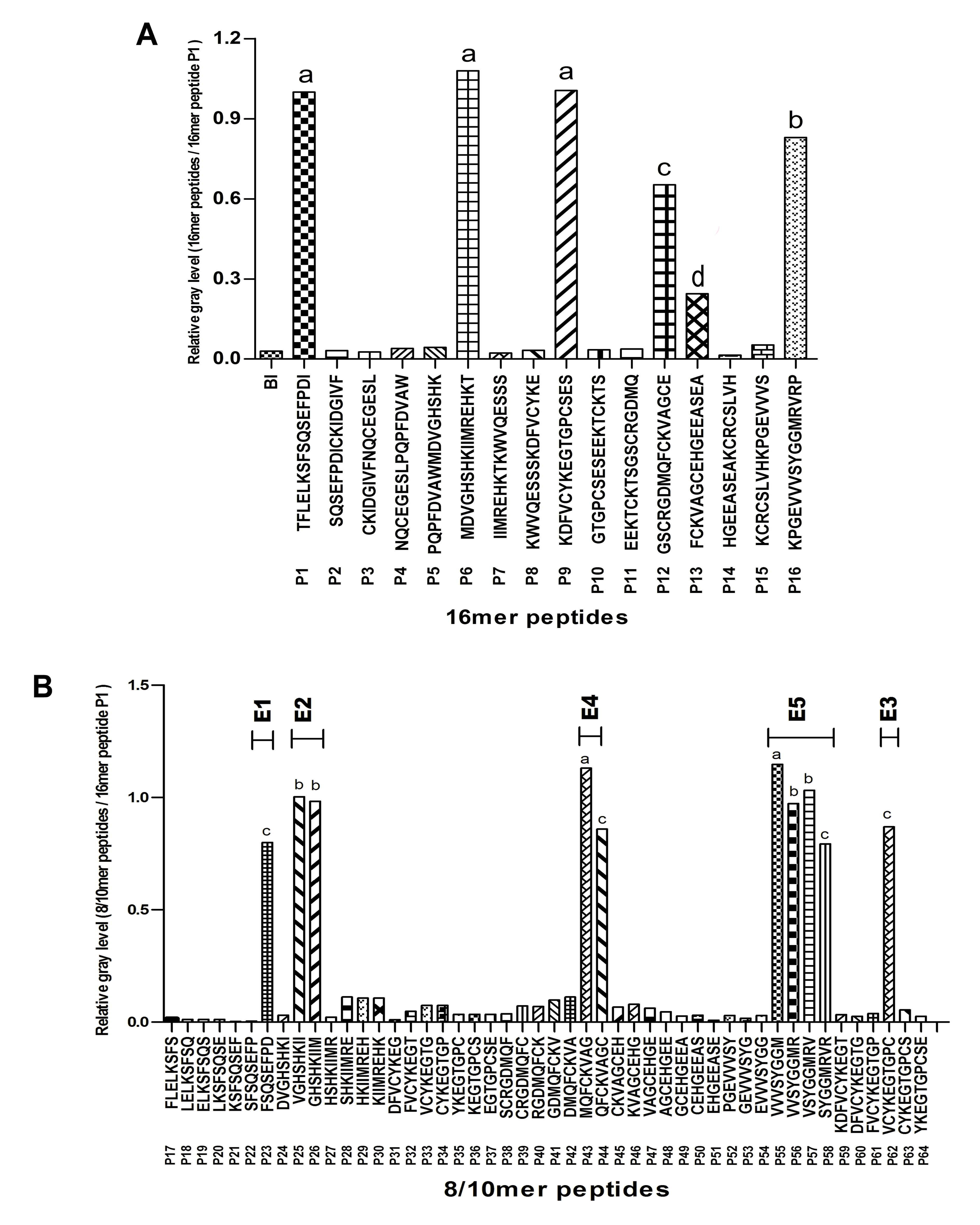

Supplement: S1 Fig — To determine the sensitivity of the antigen-antibody reaction involving the 16mer (A) and 8/10mer (B) peptides, quantitative analyses were performed using the same quantity of peptides for detection. The relative grayscale level of each 16/8/10mer peptide compared to the positive 16mer peptide P1 was analyzed according to the results in Fig 3. Statistical analysis of data was performed using one-way analysis of variance (ANOVA) to determine the significant differences using SPSS software. Letters (a, b, c) indicate the significant differences (P<0.05). (TIF) [file pone.0248005.s003.tif]
