# Supplementary material for: Fine mapping epitope on Glycoprotein-Gn from Severe Fever with Thrombocytopenia Syndrome Virus
Source: PLoS One. 2021 Mar 2;16(3):e0248005. doi: 10.1371/journal.pone.0248005 (PMC7924767; doi:10.1371/journal.pone.0248005)
Supplement: S1 Raw images — (PDF) [file pone.0248005.s004.pdf]

Raw Figure 2

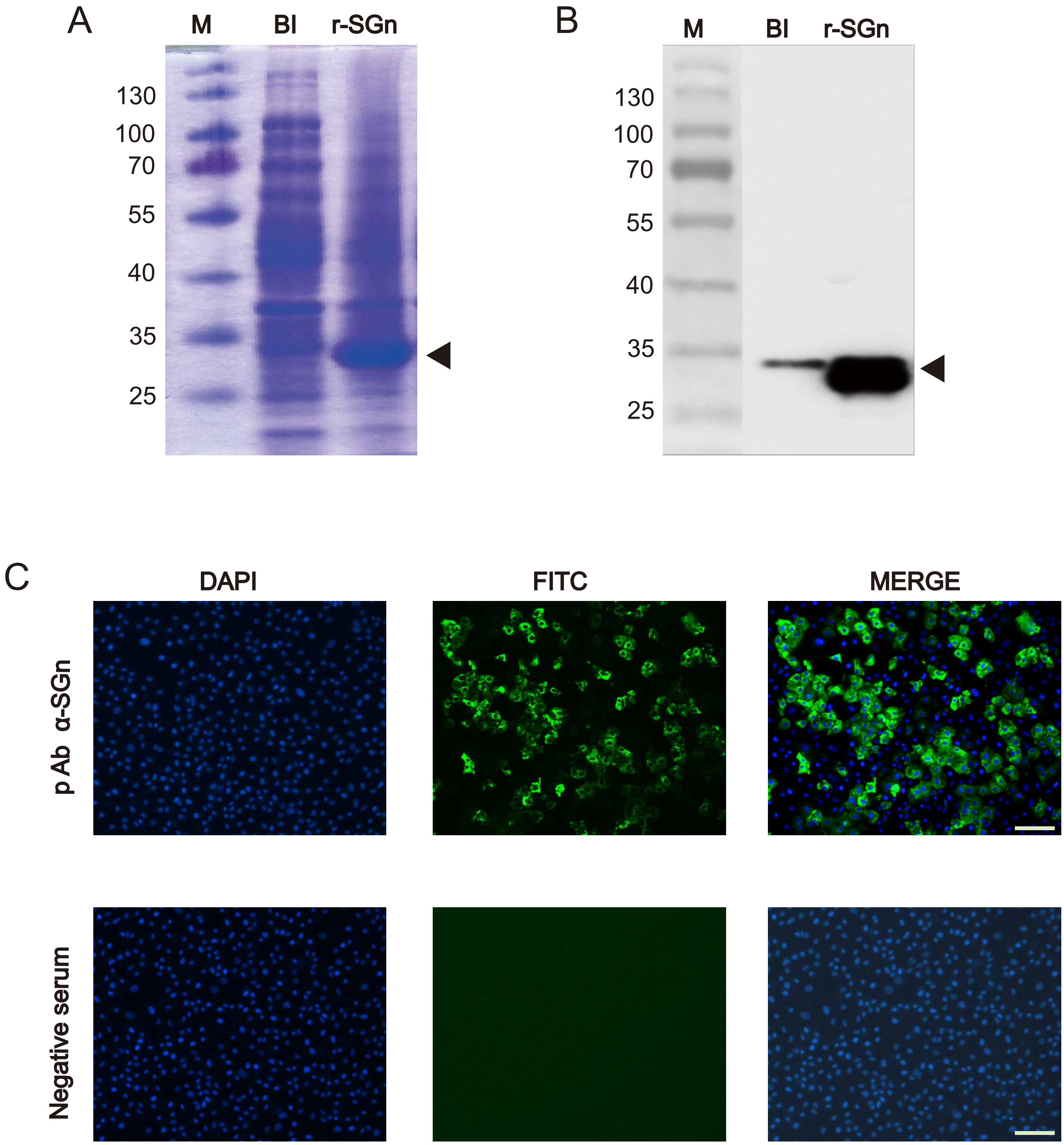

Fig 2. SDS-PAGE and Western blot analysis of r-SGn expression and identification of reactivity of rabbit pAb α-SGn. (A) SDS-PAGE analysis of r-SGn expression. M: standard protein marker; BI, total bacterial protein before IPTG induction. (B) Identification of the antigenicity of r-SGn by Western blot using rabbit pAb α-SGn. (C) Identification of the reactivity of pAb α-SGn with the viral particle in Vero cells by IFA; bars indicate 100 μm.

Raw Figure 3

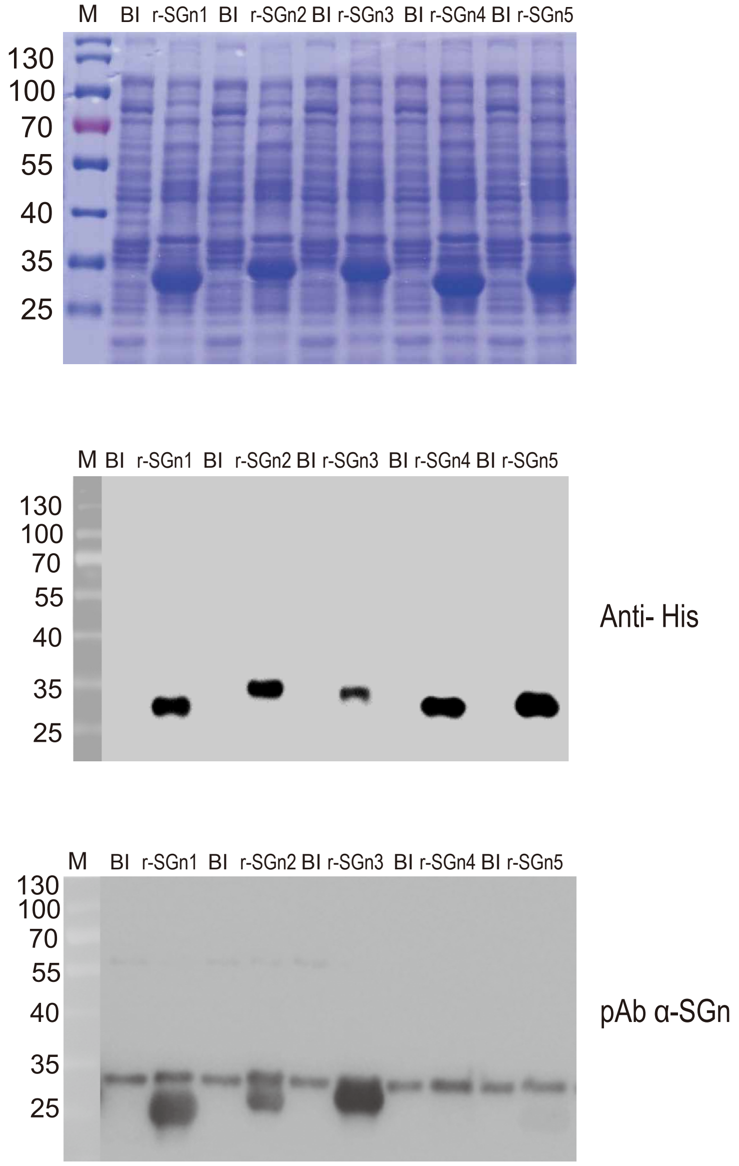

Fig 3. Prokaryotic expression and immunoblot analysis of truncated SGn segments. SDS-PAGE and Western blot analysis of expressed r-SGn1, r-SGn2, r-SGn3, r-SGn4, and r-SGn5 using rabbit pAb α-SGn. BI, total bacterial protein before IPTG induction. The arrows represent the three expressed target segments on the gel and the reactive segments in the Western blot analysis. Samples were loaded on the same gels and were processed in parallel.

Raw Figure 4A

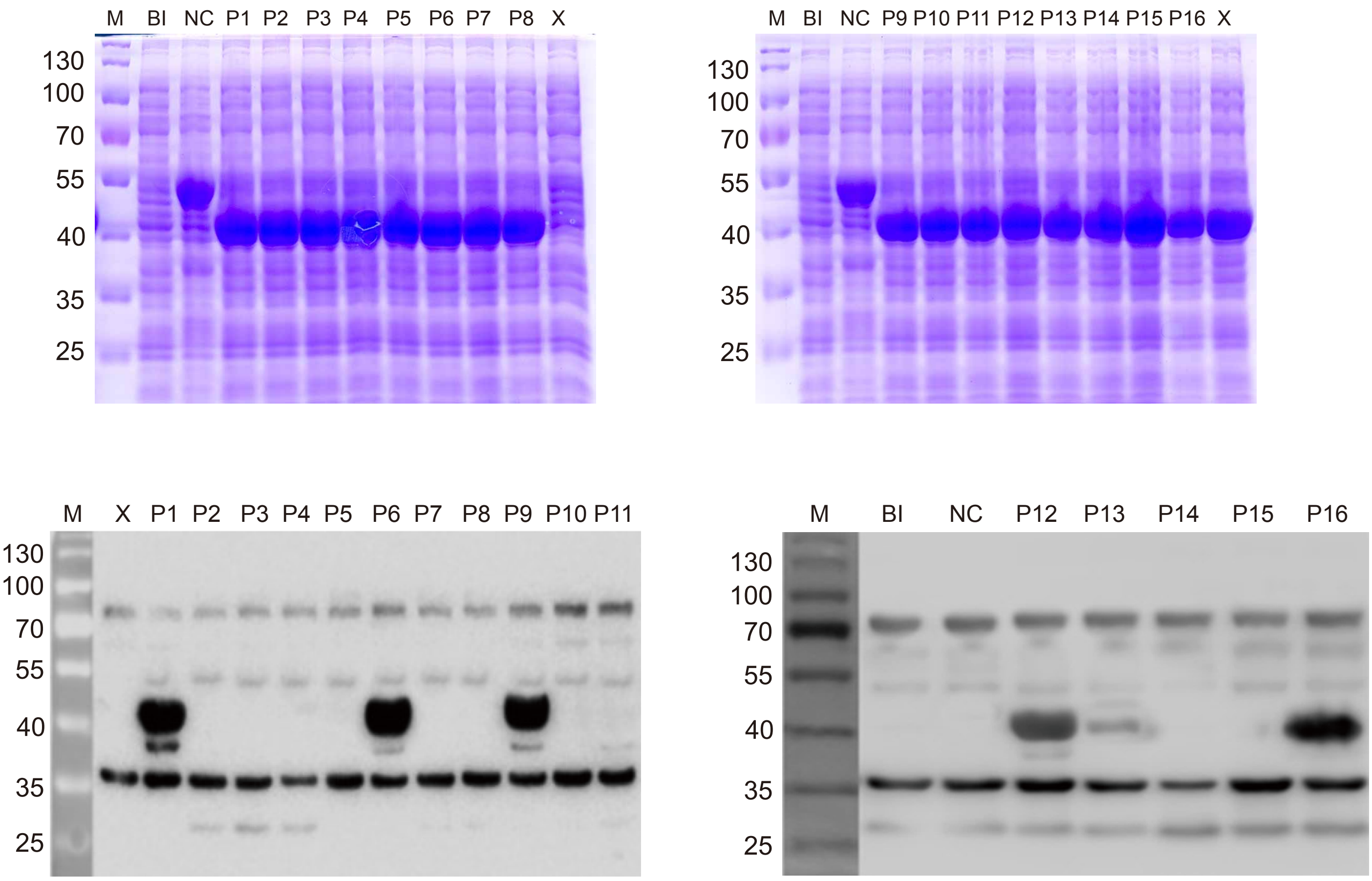

Fig 4. SDS-PAGE and Western blot analysis of MBP fusion proteins expressed 16/8/10mer peptides derived from SGn.

(A) Western blot analysis of expressed 16mer peptides using rabbit pAb α-SGn. BI, total bacterial protein before IPTG induction. NC, negative control (MBP protein expressed by pMAL-c2x).

Raw Figure 4B

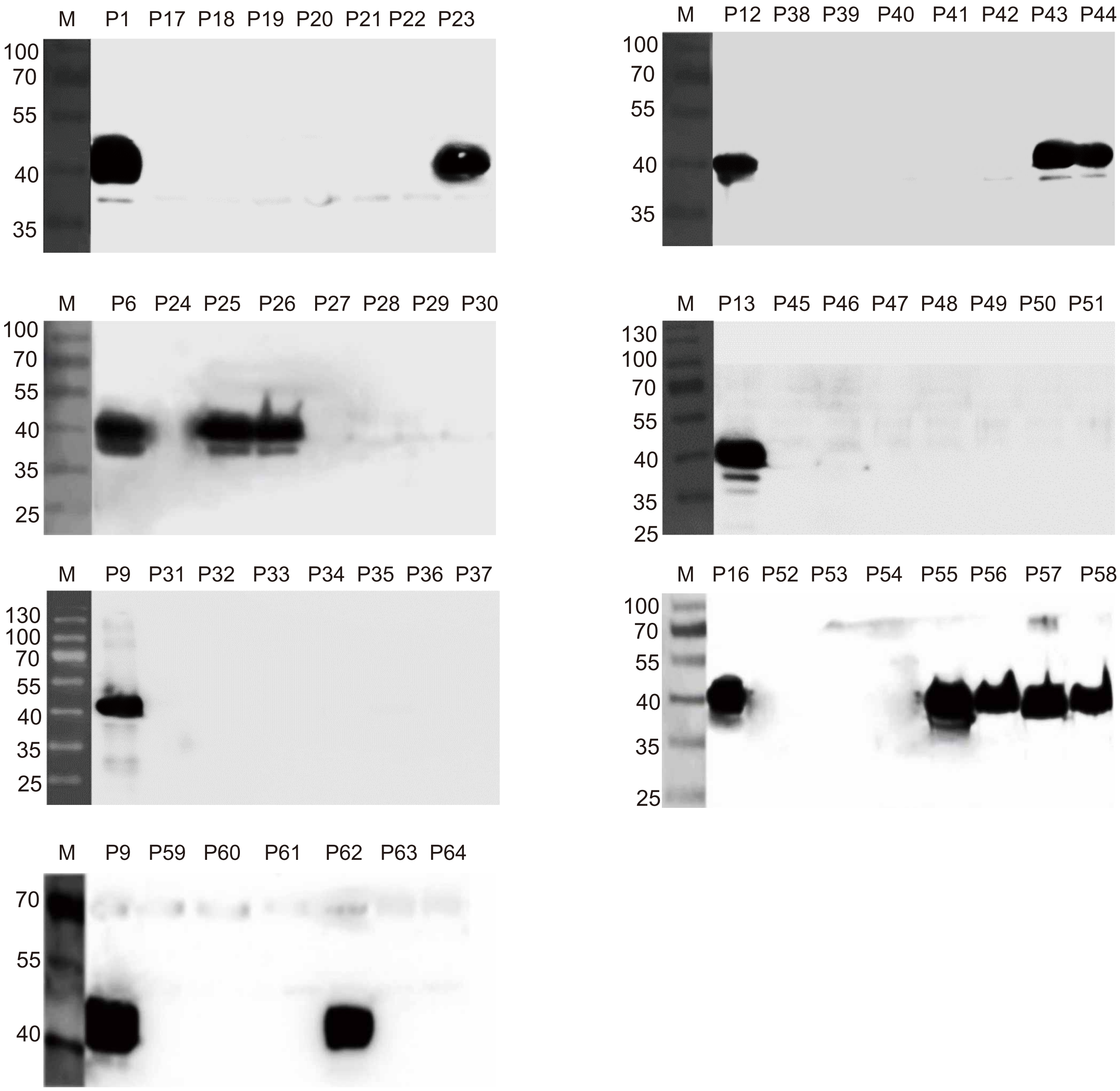

Fig 4. SDS-PAGE and Western blot analysis of MBP fusion proteins expressed 16/8/10mer peptides derived from SGn.
(B) Western blot analysis of 42 expressed 8mer peptides and 6 expressed 10mer peptides.

Raw Figure 6

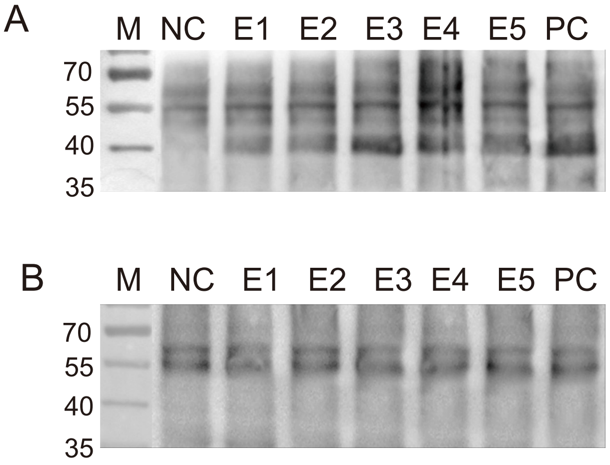

Fig 6. Western blot of five 8/10mer peptides containing identified epitopes performed using positive sera from sheep with a confirmed history of SFTSV infection. (A) A positive serum sample from sheep with a confirmed history of SFTSV infection. (B) A serum sample from sheep with no history of SFTSV infection was used as a negative control. NC, negative control (MBP protein expressed by pMAL-c2x). PC, positive control (16mer peptide P1 recognized by sheep positive serum).
