# Supplementary material for: Fine mapping epitope on Glycoprotein-Gn from Severe Fever with Thrombocytopenia Syndrome Virus
Source: PLoS One. 2021 Mar 2;16(3):e0248005. doi: 10.1371/journal.pone.0248005 (PMC7924767; doi:10.1371/journal.pone.0248005)
Supplement: Attachment — Submitted filename: Response to Reviewers.docx [file pone.0248005.s005.docx]

Response to Reviewers

Dear Editor Dr. Paulo Lee Ho,

Thank you very much for your letter and advice. We have revised the manuscript entitled “Fine mapping epitope on Glycoprotein-Gn from Severe Fever with Thrombocytopenia Syndrome Virus”, and we would like to re-submit it for your consideration. We also appreciate all the critical comments from you and reviewers. With these improvements, we hope that the current version can meet the Journal’s standards for publication. The following is a point-by-point response to all those comments and a list of changes we have made to the manuscript. We have addressed the comments raised by the reviewers, and the amendments are highlighted in red in the revised manuscript. Point by point responses to the reviewers’ comments are listed below in this letter, and the responses are highlighted in blue.

We would like to express our sincere thanks to the reviewers for the constructive and positive comments.

I look forward to hearing from you soon.

With best wishes,

Yours sincerely,

Surong Sun, Prof.

Xinjiang Key Laboratory of Biological Resources and Genetic Engineering, College of Life Science and Technology, Xinjiang University, Urumqi 830046, PR China

E-mail: sr_sun2005@163.com Tel/Fax: 086-991-8582554

**Editor Comments to the Author:**

Thank you for submitting your manuscript to PLoS One. After careful consideration, we feel that it has merit but does not fully meet PLoS One’s publication criteria as it currently stands. Therefore, we invite you to submit a revised version of the manuscript that addresses the points raised during the review process.

1) Please provide more details of the antiserum used to map the Severe Fever with Thrombocytopenia Syndrome Vírus Glycoprotein. How was it generated? What is it specificity? Was is purified? How?

**Response:** Thanks for your suggestion. We have provided information on antiserum used to map the Severe Fever with Thrombocytopenia Syndrome Vírus Glycoprotein Gn in the materials and methods section in line 94-102.

2) The authors did not show that the linear epitopes are protective or induce neutralizing antibodies against the vírus. Modify the discussion accordingly;

**Response:** Thanks for the kind remind. We added “However, we have not yet known that the identified five BCEs of SGn in this study will obtain the neutralizing activity. Further study in this direction is needed for verification.” in the discussion section in line 374-376.

3) Please, see the comments raised by both the reviewers.

**Response:** Thanks. We have revised the manuscript according to the reviewer comments.

**Journal Requirements:**

When submitting your revision, we need you to address these additional requirements.

1. Please ensure that your manuscript meets PLoS One's style requirements, including those for file naming. The PLoS One style templates can be found at https://journals.plos.org/plosone/s/file?id=wjVg/ PLoS One _formatting_sample_main_body. pdf and

https://journals.plos.org/plosone/s/file?id=ba62/ PLoS One _formatting_sample_title_ authors _affiliations.pdf

**Response:** Thanks for remind. We have adapted the format as PLoS One's style requirements in the revised manuscript.

2. In your Methods section, please specify the source of the serum samples from the healthy rabbit and healthy sheep used for your study.

**Response:** Thanks for your suggestion. We specified the source of the serum samples from the healthy rabbit and healthy sheep used for our study in 102-107. The sheep serum samples used in the study were kindly provided by Professor Yujiang Zhang from Xinjiang Centers for Disease Control and Prevention (XJCDC). The SFTSV positive or negative sheep serum samples were previously identified using an immunofluorescence assay (IFA) and enzyme-linked immunosorbent assay (ELISA) [27]. SFTSV negative sheep serum and non-immunized New Zealand rabbit serum were used as negative controls in the Western blot and IFA, respectively.

[27].	Moming A, Zhang YJ, Chang CC, Yu H, Wang MF, Hu Z, et al. Antigenicity of severe fever with thrombocytopenia syndrome virus nucleocapsid protein and its potential application in the virus serodiagnosis. Virol Sin. 2017 Feb; 32(1): 97-100. https://doi: 10.1007/s12250-016-3928-9 PMID: 28120219

3. We suggest you thoroughly copyedit your manuscript for language usage, spelling, and grammar. If you do not know anyone who can help you do this, you may wish to consider employing a professional scientific editing service.

**Response:** Thanks for the kind suggestion. This manuscript was edited for correct English language usage, grammar, punctuation and spelling by qualified native English speaking editors at The American Editor Group (<https://www.mjeditor.com/>), and the corrections of language usage, spelling, and grammar are highlighted in blue in the revised manuscript. We hope that the revised version of the manuscript is now acceptable for publication in your journal.

4. PLOS ONE now requires that authors provide the original uncropped and unadjusted images underlying all blot or gel results reported in a submission’s figures or Supporting Information files. This policy and the journal’s other requirements for blot/gel reporting and figure preparation are described in detail at https://journals.plos.org/plosone/s/figures#loc-blot-and-gel-reporting-requirements and https://journals.plos.org/plosone/s/figures#loc-preparing-figures-from-image-files. When you submit your revised manuscript, please ensure that your figures adhere fully to these guidelines and provide the original underlying images for all blot or gel data reported in your submission. See the following link for instructions on providing the original image data: <https://journals.plos.org/plosone/s/figures#loc-original-images-for-blots-and-gels>.

**Response:** Thanks for the suggestion. According to the requirements of PLoS One, we have

provided the original uncropped and unadjusted images underlying all blot or gel results reported in a submission as “S1_raw_images.pdf” file.

5. We note that you have included the phrase “data not shown” in your manuscript. Unfortunately, this does not meet our data sharing requirements. PLoS does not permit references to inaccessible data. We require that authors provide all relevant data within the paper, Supporting Information files, or in an acceptable, public repository. Please add a citation to support this phrase or upload the data that corresponds with these findings to a stable repository (such as Figshare or Dryad) and provide and URLs, DOIs, or accession numbers that may be used to access these data. Or, if the data are not a core part of the research being presented in your study, we ask that you remove the phrase that refers to these data.

**Response:** Thanks for the suggestion. We have removed the inaccessible data and phrases about“data not shown”.

**Review Comments to the Author:**

Reviewer #1: Moming A et al. have mapped the epitopes on glycoprotein Gn of SFTSV. The study is interesting and the work was done very carefully and very well. There are few mistakes need to be changed in the manuscript.

1.	Throughout the manuscript Western should be capitalized for Western blot.

**Response:** Thanks for the good remind. We have revised Western to capitalized Western blot in the manuscript.

2.	The following method description in the result section should be deleted.

1)	Between lines 221 and 224, “Expressed recombinant SGn (r-SGn) was first separated by SDS-PAGE (Fig 2A) and then transferred to nitrocellulose membranes; pAb α-SGn (1:1000 dilution) was used as the primary antibody, and HRP-conjugated goat anti-rabbit IgG (1:5000 dilution) was used as the secondary antibody”.

2)	Between lines 257 and 259, “NC membranes were blocked with 5% (w/v) skimmed milk powder, incubated sequentially with rabbit pAb α-SGn (1:1000 dilution), and incubated with goat anti-rabbit (1:5000 dilution), and then visualized by ECL”.

3)	Between lines 277 and 279, “NC membranes were blocked with 5% (w/v) skimmed milk powder, incubated sequentially with rabbit pAb α-SGn (1:1000 dilution) and goat anti-rabbit IgG (1:5000 dilution), and then visualized by ECL”.

Response: Thanks for the kind suggestion. We have deleted the above method description in the result section in the revised manuscript.

3. Missing references:

1) Online 59, a reference (Yu XJ, Liang MF, Zhang SY, et al. Fever with thrombocytopenia associated with a novel bunyavirus in China. N Engl J Med. 2011;364(16):1523-1532) should be added.

**Response:** Thanks for the valuable suggestion. We have added this reference in line 37.

.

2) On line 65, SFTSV is not Banyangvirus. You need change the name of the virus to Bandavirus and use the reference “Kuhn JH, Adkins S, Alioto D, et al. 2020 taxonomic update for phylum Negarnaviricota (Riboviria: Orthornavirae), including the large orders Bunyavirales and Mononegavirales. Arch Virol. 2020;165:3023-3072” to replace reference 12.

**Response:** Thanks for the good remind. We apologize for this oversight, the classification of SFTSV was updated according to reference (14) as *Bandavirus* genus within the family *Phenuiviridae*. Reference [12] has been replaced as [14] in the revised version in line 44.

[14] Kuhn JH, Adkins S, Alioto D, Alkhovsky SV, Amarasinghe GK, Anthony SJ, et al. 2020 taxonomic update for phylum Negarnaviricota (Riboviria: Orthornavirae), including the large orders Bunyavirales and Mononegavirales. Arch Virol. 2020 Dec;165 (12): 3023-3072. https://doi: 10.1007/s00705-020-04731-2 PMID: 32888050

3) On line 65, you need add references for the statement “Studies have indicated that GP mediate the first step in the virus replication cycle viral entry, and are the only targets for neutralizing antibodies”.

**Response:** Thanks for the good remind. We apologize for our earlier lack of clarity. We have added references [15] and [16] in line 48 of the revised manuscript.

[15]	Tani H, Shimojima M, Fukushi S, Yoshikawa T, Fukuma A, Taniguchi S, et al. Characterization of glycoprotein-mediated entry of severe fever with thrombocytopenia syndrome virus. J Virol. 2016 May 12; 90(11): 5292-5301. https://doi: 10.1128/JVI.00110-16 PMID: 26984731

[16]	Hofmann H, Li X, Zhang X, Liu W, Kühl A, Kaup F, et al. Severe fever with thrombocytopenia virus glycoproteins are targeted by neutralizing antibodies and can use DC-SIGN as a receptor for pH-dependent entry into human and animal cell lines. J Virol. 2013 Apr; 87(8): 4384-4394. https://doi: 10.1128/JVI.02628-12 PMID: 23388721

Reviewer #2: The manuscript entitles “Fine mapping epitope on Glycoprotein-Gn from Severe Fever with Thrombocytopenia Syndrome Virus” describes the linear epitope mapping of Gn protein of SFTS virus. The authors showed the five linear epitopes in the Sn protein, 196-203, 232-238, 256-265, 285-290, and 316-320 amino acid residues, respectively. The epitopes were mapped using rabbit anti Sgn antibody and the epitopes were also recognized by SFTS antibody-positive sheep sera. The manuscript is well written and the methods to map the epitopes were appropriate. However, there are two major criticism as written in the “Major points”.

Major points:

The epitopes were mapped using rabbit pAb a-SGn (reference No8) supplied from Prof. Deng, however in the reference manuscript any detail of the rabbit a-SGn was written. Thus, the authors should describe the detail of the antibody as to how the rabbit serum was prepared, whether the antibody has viral neutralizing antibody against SFTSV.

**Response:** Thanks for the good remind. We have provided information on antiserum used to map the Severe Fever with Thrombocytopenia Syndrome Vírus Glycoprotein Gn in the materials and methods section in line 94-102. The main preparation methods are as follows：The coding region (aa, 189-451) of SFTSV-Gn from strain WCH/97/HN/China/2011 was amplified by PCR using 2×Rapid Taq Master Mix (Vazyme Biotech, Nanjing, China) according to the manufacture’s instruction. The PCR products were cloned into the plasmid pET-28a to generate the expression plasmid pET-28a-*SGn* and the insert was confirmed by sequencing. Protein expression and purification were conducted as described [27]. New Zealand rabbits were injected intramuscularly with 0.5 mg of purified SGn segment and immunized at two-week intervals according to the conventional animal immune method. After the third immunization for two weeks, rabbit antiserum was separated and stored at -80℃ until use. The neutralizing activity of rabbit pAb α-SGn against SFTSV was detected using IFA, but no neutralizing effect was found.

[27] Moming A, Zhang YJ, Chang CC, Yu H, Wang MF, Hu Z, et al. Antigenicity of severe fever with thrombocytopenia syndrome virus nucleocapsid protein and its potential application in the virus serodiagnosis. Virol Sin. 2017 Feb; 32(1): 97-100. https://doi: 10.1007/s12250-016-3928-9 PMID: 28120219

From the data shown in the manuscript, it is too much to discuss “these linear epitopes identified in the manuscript provide fundamental data for the elucidation of the design and development of a SFTSV multi-epitope peptide vaccine”, since the authors never showed the antibodies to these linear epitopes have viral neutralizing capacity of not. Actually, the authors describe “However, whether the identified five BCEs of SGn in this study have neutralizing activity require further verification (line 395-396). The reviewer agrees that the data shown in the manuscript provide useful data for the development of a novel SFTS antibody detection antigen. Thus, the authors are recommended to remove the sentence describing vaccine development.

**Response:** Thanks for the good suggestion. We have removed the sentence describing vaccine development in the revised manuscript.

Minor points;

Line 62: “including goats, cattle, dogs, and chickens [8-10]” lacks cats as susceptible animal. Scientific Reports, 19(1): 11990 (2019) showed that cats were susceptible for SFTSV and the virus caused lethal infection in cats. The authors should add this manuscript to the reference.

**Response:** Thanks for the kind remind and suggestion. We have added cats as susceptible animal for SFTSV, and added as reference [12] in line 40 in the revised manuscript.

[12] Park ES, Shimojima M, Nagata N, Ami Y, Yoshikawa T, Iwata-Yoshikawa N, et al. Severe fever with thrombocytopenia syndrome Phlebovirus causes lethal viral hemorrhagic fever in cats. Sci Rep. 2019 Aug 19; 9(1): 11990. https://doi: 10.1038/s41598-019-48317-8 PMID: 31427690

Line 110: “involving” should be “involve”

**Response:** Thanks for the good remind. “involving” was revised as “involve” in line 90.

Line 142-143: “computer controlled microscope” should be changed to “ the name of microscope product name”

**Response:** Thanks for the kind remind. “computer controlled microscope” was changed as “IX73 microscope” in lines 129.

Line 143: “Olympics” is “Olympus” ??

**Response:** Thanks for the good remind. “Olympics” was revised as “Olympus” in line 129.

Line 269: “ … MBP fusion expressed …” should be “… MBP fusion proteins expressed …

**Response:** Thanks for the good remind. “MBP fusion expressed” was revised as “MBP fusion proteins expressed” in line 250.

Line 364 -365: “mAb 4-5, a neutralizing antibody identified in SFTS recovered patients” is not precise. mAb 4-5, a neutralizing monoclonal ScFv antibody cloned from SFTS recovered patient.”

**Response:** Thanks for the good remind. “mAb 4-5, a neutralizing antibody identified in SFTS recovered patients” was revised as “mAb 4-5, a neutralizing monoclonal single chain fragment variable (ScFv) antibody cloned from SFTS recovered patient” in lines 342-343.
