# Supplementary material for: Fine mapping epitope on Glycoprotein-Gn from Severe Fever with Thrombocytopenia Syndrome Virus
Source: PLoS One. 2021 Mar 2;16(3):e0248005. doi: 10.1371/journal.pone.0248005 (PMC7924767; doi:10.1371/journal.pone.0248005)
Supplement: Attachment — Submitted filename: Response to Reviewers.docx [file pone.0248005.s006.docx]

Response to Reviewers

Dear Editor Dr. Paulo Lee Ho,

Thank you very much for your letter and advice. We have revised the manuscript entitled “Fine mapping epitope on Glycoprotein-Gn from Severe Fever with Thrombocytopenia Syndrome Virus”, manuscript number: PONE-D-20-32530R1. We would like to re-submit it for your consideration. We have addressed the comments raised by the reviewers, and the amendments are highlighted in red in the revised manuscript. Responses to the reviewers’ comments are listed below in this letter, and the responses are highlighted in blue.

We would like to express our sincere thanks to the reviewers for the constructive and positive comments.

We look forward to hearing from you soon.

With best wishes,

Yours sincerely,

Surong Sun, Prof.

Xinjiang Key Laboratory of Biological Resources and Genetic Engineering, College of Life Science and Technology, Xinjiang University, Urumqi 830046, PR China

E-mail: sr_sun2005@163.com Tel/Fax: 086-991-8582554

**Review Comments to the Author:**

Reviewer #1: The minor comments are on line 178 bacteria culture should be bacterial culture and on line 242 E. coli should be italicized.

**Response:** Thanks for the good remind. We apologize for our carelessness, we have revised “bacteria culture” as “bacterial culture” on line 178, and italicized E. coli on line 242 in the manuscript.
